# Address to the Graduating Class of the Ohio Dental College

**Published:** 1868-02

**Authors:** Geo. W. Kelly

**Affiliations:** President of the Board of Trustees


### ADDRESS TO THE GRADUATING CLASS OF THE OHIO DENTAL COLLEGE.

BY DR. GEO. W. KELLY, PRESIDENT OF THE BOARD OF TRUSTEES.

GENTLEMEN:—A quarter of a century since, before the birth of the " Ohio College of Dental Surgery, (our honored *alma mater*,) after a pupilage of nearly three years, we said good-bye, to a much loved and respected preceptor. Being armed with the necessary instruments and material, started out to make a beginning in the battle for professional success.

Our preceptor had a lucrative practice, and in the ardor of youthful enthusiasm we flattered ourself that we had rendered him important assistance, in mastering many and very difficult operations. Fully impressed with *this* idea, and picturing out an easily acquired practice, we could not see *any* conceivable reason why abundant success should not crown our first efforts; little dreaming of the trials and perplexities that followed close upon us. Years of labor, with an earnest desire for the elevation of our chosen specialty, brings with them the unalterable conclusion, that there is *yet* an *immense* amount to be learned; and, to-day, feel we know less than we supposed we could master at our commencement.

This, gentlemen graduates, is your commencement. *It is*, we believe, almost a universal mistake made by the majority of young men, who, after going through the prescribed routine of study and labor, preparatory to entering the profession of Dentistry, Medicine, Divinity, Law, or in pursuit of the Mechanic Arts, with a diploma or other recommendation, that a location is chosen, and services at once offered to the public, with the assurance that "they know it all." Young gentlemen, if any of *you* have allowed this erroneous conclusion to find a lodgment in your minds, be at *once* undeceived. Professional success, with a lucrative practice, is gained only by incessant labor These diplomas, which you receive to-night, are only the indorsement of the authorities of the "Ohio College of Dental Surgery," as to your being duly qualified to commence your professional career. It would, indeed, be a sad misfortune and fatal to your future success, did you imagine that while a pupil in this institution you had been taught all the necessary requirements, and with the close of your pupilage you were no longer to be a diligent student.

During the long months you have spent within these walls, you have acquired the habit of study, which should still be adhered to. According to an old maxim, "habit becomes a second nature," rendering labor easy, the performance of duty a pleasure.

Habit will guard you against the influence of bad example, and protect you from unforeseen temptation. We would therefore urge upon you particularly, the importance of cultivating intellectual habits. Not only will you be personally benefited, but render yourselves worthy of high and honorable recognition, by the "shining lights" in the profession, and refined society. During your first years of practice, you will doubtless have leisure hours, which can be profitably spent in pursuing the study of Anatomy, Physiology, Pathology, Chemistry, Dental Medicine, Surgery and the other sciences, which, during your pupilage you have comparatively but just begun; all offering boundless fields for thought and investigation to the student whose ambition urges him on to qualify himself in the best manner possible for usefulness and honor in his profession.

There is no nook or corner for the indolent man, we are marching rapidly onward, and the sooner such drones are expelled from the dental hive, and seek some other calling, the better it will be for all interested.

Dental science has made rapid strides within the present century, and is acknowledged as a specialty of general medicine and surgery, requiring an extensive range of knowledge for success in practice.

Every year adds momentum to its onward march, and if you hope to keep up with your brethren in the race for professional renown, you must industriously use and profit by every means within your power, and not be content until you have acquired a thorough knowledge of all that is most intimately connected with your calling.

It is said, "There is no royal road to knowledge," which is certainly true in our profession, for it can only be reached by habitual diligence.

We congratulate you, young gentlemen, that you are entering the profession under such auspicious circumstances. You can build on the foundation laid by your predecessors after long years of investigation and labor.

With this on which to build, it will be your duty and we trust your highest aim, to beautify and adorn the structure as it rises higher and higher. As they have toiled unweariedly and uncomplainingly, so may you never feel like giving up, even though at times all should look dark; remember, "every cloud has a silver lining," and go manfully on with the work.

We do not expect you to fill up *all* your time with work, you should, of course, keep generally posted in the literature and news of the day, that you may be an ornament to society, socially and politically.

Not least in importance is our Dental literature; it should have a prominent place in your affections, and should you unfortunately have a neighbor who is not a subscriber (truly an object of commiseration), see that he becomes one, that he may be "brought from darkness to light." You will lose nothing by your efforts to elevate those who would drag you down, by bringing reproach on the profession.

Guard well its reputation by giving encouragement to none but the truly worthy to enter its portals, requiring a prescribed term of pupilage, and a regular course in some Dental college.

In regard to Dental societies, it would be impossible to estimate the advantages arising from a regular attendance, whenever circumstances will permit. Meet your brethren with a kind, friendly feeling; encouraging them by your presence of a desire for improvement and readiness to impart any information you may possess that would be of advantage. They dissipate personal discords and jealousy.

By social contact the discussion of the various subjects of interest, and comparison of the different modes of practice, each one is stimulated to aim at a greater degree of perfection in all his operations.

They have an elevating influence, and after every meeting you will love your profession and brethren more than ever; be a better man, and also a better Dentist.

Should any of your competitors not be a member (and strange as it may seem there are some good men who are not), urge him with a determination that knows no failure, to become one; and my word for it, he will ever after bless you.

We advise you not to be indifferent to the importance of cultivating friendly relations with the members of the Medical profession, as you will frequently meet them in consultation, in which you will have ample opportunity to show your ability in deciding as regards the merits of the case, so far as your services are required.

Lest I weary you with further remarks, I will close, rejoicing with you that your examinations have been so satisfactory, and that you are duly qualified to receive these tokens of our confidence and regard.

But in the midst of our rejoicings and congratulations, at this time, when *we* are about to part, will it not be well to remember kindly though with sorrow, him, who parted from us during the past session, and went away into the "dark valley of the shadow of death?" It becomes us here, to drop tears of affection to the memory of your beloved classmate, whose *hopes*, ambitions and prospects were as bright, and whose future seemed as certain as yours at the beginning of this session, but whose earthly career was so suddenly cut short by the grim visitor, who must sooner or later knock at each of our doors. We know not the day nor the hour, but let us be found as he was, in the ranks, fighting the battle of life with *our* best energies; making the most of time allotted us, not as though it were our all, but as a part of an eternity that belongs to each of us.

Gentlemen Graduates,—By the authority committed to me as President of the Board of Trustees, it is a pleasurable duty to confer on each of you this Diploma of "Doctor of Dental Surgery." The Faculty in conferring these marks of distinction and merit, do so, fully believing that you will devote your energies to the good of the profession and mankind.

In connection with these, and as a parting gift, the Faculty present to each of you a copy of the "Holy Bible," which is of all books the purest and most "venerably good." May its precepts ever guide you; and in all your transactions, social and professional, keep before you the golden rule,—"Do unto others, as ye would they should do unto you."

